# THE SOCIOLOGY OF AGING AND SOCIAL GERONTOLOGY: CRITICAL TENSIONS AND NECESSARY DISTINCTIONS

**DOI:** 10.1093/geroni/igad104.2182

**Published:** 2023-12-21

**Authors:** Paul Higgs, Chris Gilleard

**Affiliations:** University College London, London, England, United Kingdom; University College London, London, England, United Kingdom

## Abstract

This paper argues that the sociology of ageing has been diverted from developing a clearly sociological approach to ageing and old age by its entanglement with social gerontology. Dominated by social and health policy and advocacy on behalf of older people, the sociological analysis of later life has taken second place. The rise of critical gerontology and cultural gerontology have put further distance between sociology and the sociology of ageing. The changes to ageing and old age challenge the residualisation of ageing within the wider sociological community and set up new tasks for a sociology of ageing which resonates with contemporary later life. We argue that to achieve those tasks requires not only separating the sociology of ageing from social gerontology, but also employing contemporary sociological theory can enable the sociology of ageing to move beyond the ‘problem’ of old age. This involves an analysis of the social structural dimensions of contemporary ageing, its symbolic representations and the embodied and agentic experiences of people becoming older. We conclude that a re-envisioned sociology of ageing can also better equip social gerontology with more effective tools to understand the ‘new ageing’.

